# Cardiovascular Profile and Cardiovascular Imaging After Bariatric Surgery: A Narrative Review

**DOI:** 10.3390/medicina61010073

**Published:** 2025-01-04

**Authors:** Mihaela Toader, Liliana Gheorghe, Costin Chirica, Ionuț-Alexandru Ghicu, Sabina-Ioana Chirica, Andreea Isabela Mazga, Danisia Haba, Mădălina Maxim, Ancuța Andreea Miler, Daniela Crișu, Mihai Ștefan Cristian Haba, Daniel Vasile Timofte

**Affiliations:** 1 Doctoral School, “Grigore T. Popa” University of Medicine and Pharmacy, 16 Universitatii Str., 700115 Iasi, Romania; mihaela.toaderr@gmail.com (M.T.); chiricasabinaioana@gmail.com (S.-I.C.); madalynamaxim@yahoo.com (M.M.); miler.ancuta@umfiasi.ro (A.A.M.); 2 Department of Radiology, “Grigore T. Popa” University of Medicine and Pharmacy, 700115 Iasi, Romania; 3 Faculty of General Medicine, “Grigore T. Popa” University of Medicine and Pharmacy, 700115 Iasi, Romania; ghicualex2005@gmail.com; 4 Faculty of General Medicine, Iuliu Hatieganu University of Medicine and Pharmacy, 400012 Cluj-Napoca, Romania; 5 Department of Oral and Maxillofacial Surgery, Faculty of Dental Medicine, “Grigore T. Popa” University of Medicine and Pharmacy, 16 Universitatii Str., 700115 Iasi, Romania; danihaba@yahoo.com; 6 Department of Surgery, “St. Spiridon” County Clinical Emergency Hospital, 700111 Iasi, Romania; 7 Cardiology Clinic, “St. Spiridon” County Clinical Emergency Hospital, 700111 Iasi, Romania; daniellacrisu@yahoo.com (D.C.); cristi.haba@gmail.com (M.Ș.C.H.); 8 Department of Internal Medicine I, Faculty of Medicine, “Grigore T. Popa” University of Medicine and Pharmacy, 700115 Iasi, Romania; 9 Department of Surgery, “Grigore T. Popa” University of Medicine and Pharmacy, 700115 Iasi, Romania; daniel.timofte@umfiasi.ro

**Keywords:** bariatric surgery, cardiovascular structure, cardiovascular function

## Abstract

*Background and Objectives*: Up until now, behavioral interventions and pharmacological therapies were the main approach available for the management of obesity. Diet and exercise, when used as a singular therapeutic method, are inadequate for a successful outcome. Research shows promising results for the surgical treatment of obesity, especially in the area of bariatric surgery (BaS). The relevance of this study is the valuable analysis of the evolution of obese patients with increased cardiovascular risk. *Materials and Methods*: The patients eligible for BaS commonly suffer from multiple chronic conditions, including type 2 diabetes, obstructive sleep apnea, cardiovascular diseases, and non-alcoholic fatty liver disease. Additionally, obesity contributes to an increased probability of developing certain types of cancer, osteoarthritis, urinary incontinence, and chronic kidney disease. In this review, we focused especially on the cardiovascular status of obese patients who underwent bariatric procedures. *Results*: BaS has been found to be strongly associated with a reduced incidence of severe complications in individuals with a history of myocardial infarction (MI) and severe obesity. Specifically, this procedure is linked to a lower occurrence of major adverse cardiovascular events and a decrease in overall mortality. Also, BaS is correlated with a reduced risk of recurrent MI and the development of new-onset heart failure. *Conclusions*: The results of BaS involve a significant amelioration of the BMI, contributing to a considerable decrease in cardiovascular risk factors and to a notable refinement in the cardiovascular structure and function.

## 1. Introduction

Obesity is a dramatic condition with a chronic and recurrent pattern, characterized by the accumulation of excess body fat and an uncontrollable body mass. According to data published in 2016 by the World Health Organization (WHO), over 650 million people globally are affected, an impressive and rapidly growing number [1,2,3,4]. Through various mechanisms, this condition is closely linked with the coexistence and exacerbation of systemic health comorbidities, and it has been associated with an elevated risk of all-cause mortality [5].

Also, obesity is the second leading cause of preventable death, following smoking. As a result, it has undeniably become a prevalent public health issue, significantly impacting individual health and overall well-being and contributing to rising healthcare costs. In 2016, obesity was associated with four million deaths per year, and it is estimated that, by 2030, more than one billion individuals globally will be affected by this condition, impacting one in five women and one in seven men [6,7,8]. The global prevalence of obesity is primarily driven by sedentary occupations, technological advancements, and the consumption of ultra-processed foods [9].

**Figure 1:**
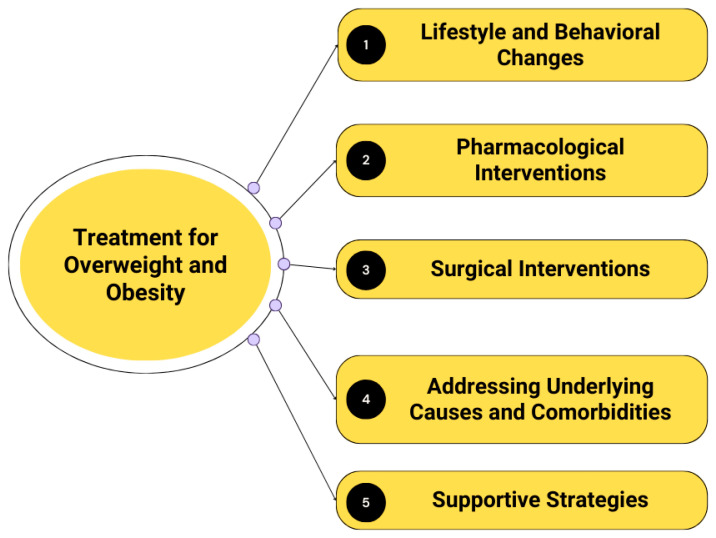
Illustrating the treatment for overweight and obesity.

The treatment of overweight and obesity involves a multifaceted approach, which aims to achieve sustainable weight loss and the improvement of overall health (Figure 1).

Up until now, behavioral interventions (including lifestyle change and nutrition) and pharmacological therapies were the main approaches available for the management of obesity. A negative energy balance that provides the basis for the treatment of overweight and obesity is part of the standard recommendation. Adherence to a dietary regimen that creates an energy deficit, leading to weight loss and subsequent maintenance of the reduced weight, regardless of the specific selected diet is a critical factor in the effective treatment of obesity. Diet and exercise, however, when used as a singular therapeutic method, are often insufficient for a successful outcome [10,11,12].

Moreover, the pharmacological treatment of obesity alone does not demonstrate any significant long-term results. The effectiveness of these treatments can vary among individuals, and long-term success often depends on the continued use of medication combined with restrictive lifestyle interventions. Progress is being made in this regard, and many innovative drugs, mainly based on the incretin effect, are currently being investigated in different phases of clinical trials [13].

Following significant progress, nowadays, an effective and long-term treatment of morbid obesity is being unfolded through the advancements of surgical procedures. The primary aim of surgical interventions for obesity is to limit the energy intake. This is achieved by restructuring the anatomy of the intestine, and therefore, these procedures modify satiety. As a result, individuals are able to consume only small quantities of food [14,15].

In this way, bariatric surgery (BaS) is widely regarded as an effective surgical intervention for achieving significant and sustained weight loss in individuals with severe obesity. Unlike approaches that rely solely on calorie restriction or exercise, BaS has demonstrated superior long-term outcomes. Its effectiveness stems from the ability to induce substantial physiological and behavioral changes that are often difficult to achieve through non-surgical methods alone. As a result, it is considered a critical treatment option for individuals who struggle to manage severe obesity through conventional strategies, and it is currently the recommended approach for this category of patients [15,16,17]. With regard to the range of surgical interventions, the Roux-en-Y gastric bypass (RYGB) and sleeve gastrectomy (SG) are the most commonly performed procedures in BaS centers worldwide [18,19].

However, the patients who are eligible for BaS commonly suffer from multiple chronic conditions, including type 2 diabetes, obstructive sleep apnea, cardiovascular diseases, and non-alcoholic fatty liver disease [20,21,22]. Specifically, in terms of systemic damage, patients with a BMI > 30 kg/m^2^ face imminent risk for cardiovascular disease (CVD). A vast range of conditions affecting the heart and blood vessels, including coronary artery disease, cerebrovascular disease, deep vein thrombosis, peripheral arterial disease, and others, are associated with a morbidly excessive body weight. Globally, CVD represents the leading cause of mortality in obese patients [23]. Data from recent literature indicate a drastically increased risk for cardiovascular events and a notably higher mortality rate in obese patients compared to those without this diagnosis [24].

Excess adiposity has been shown to adversely affect cardiovascular functions through multiple mechanisms. First of all, it generates endothelial dysfunction, which impairs the regulation of vascular tone and blood flow. Additionally, it promotes the remodeling of small blood vessels, contributing to structural and functional abnormalities in microcirculation. Adiposity also exerts toxic effects on cardiomyocytes, the specialized cells of the heart muscle, further exacerbating cardiovascular damage. Collectively, these pathological changes contribute to the development of a large range of cardiovascular conditions, including atherosclerotic and vasospastic coronary artery disease. Furthermore, these processes increase the risk of arrhythmias, cardiomyopathy, and congestive heart failure, highlighting the complex impact of excess adiposity on cardiac function and overall cardiovascular health [25].

Following surgical intervention, the cardiovascular patient profile undergoes significant changes, and while some have a favorable impact, others require additional care. Various cardiovascular risk factors, such as hypertension, diabetes mellitus, dyslipidemia, or metabolic syndrome, are investigated from both a clinical and a paraclinical perspective after surgery.

The central point of this narrative review paper is to evaluate the impact of BaS on the systemic obesity-related modifications and, more particularly, on the cardiovascular system. We chose to focus on the cardiovascular system for a thorough and extensive understanding of the outcomes of BaS. Therefore, we present the identified studies in-depth so that our research can further contribute to good practice in the management of obese patients who are candidates for BaS.

## 2. Materials and Methods

For this narrative review, we researched the literature for publications within the last 10 years. A structured search was performed on online databases, such as PubMed, Web of Science, and Scopus. Furthermore, we used historically relevant articles for a more comprehensive illustration of the concepts presented.

More precisely, for this purpose, we used a dedicated advanced search formula: (bariatric surgery OR obesity surgery OR weight loss surgery OR metabolic surgery OR Roux-en-Y Gastric Bypass OR sleeve gastrectomy) AND (cardiac imaging OR cardiac status OR cardiac dimensions OR ventricular dimensions OR cardiac function OR cardiac complications OR cardiac structure OR vascular function OR vascular structure OR vascular complications).

## 3. Clinical Evaluation of Obesity

**Figure 2:**
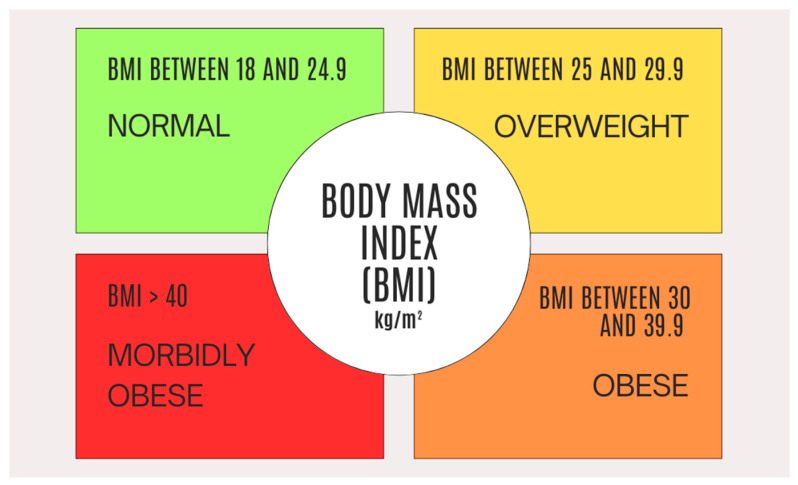
Illustrating the body mass index (BMI), a tool that healthcare providers use to estimate the amount of body fat by using mass and height measurements.

Present-day medical practice implements numerous clinical systems and standardized criteria, used to determine a person’s grade of excessive weight. In this regard, the body mass index (BMI) is still considered the most useful screening tool at an individual level worldwide. Using the BMI, obtained by calculating the mass measured in kilograms (kg) divided by the square of the height measured in meters (m^2^), we can define obesity as a BMI > 30 kg/m^2^ (Figure 2). The recommended classification of obesity according to BMI is as follows: obesity class I (BMI ranging from 30 to less than 35), obesity class II (BMI ranging from 35 to less than 40), and obesity class III (BMI of 40 or higher). The diagnosis of obesity is recommended to be based on the measurement of BMI in conjunction with a clinical evaluation of complications associated with excess weight [1,22,26,27].

Most studies correlate an increased BMI with a high cardiovascular risk. However, a controversy came to light in the specialized literature. It is known as the “obesity paradox”, and it suggests that there is an association between mildly elevated BMI and improved survival rates as well as a lower incidence of cardiovascular events in patients with chronic diseases [28].

While it is true that the BMI is a widely used tool, there are unfortunately limitations to this clinical instrument. Thus, BMI does not consider the variations in muscle mass, fat distribution, or overall body composition, which may result in the misclassification of an individual’s weight status. However, other clinical tools for the management of overweight patients have also been integrated in order to reach an increased performance. These include the measurement of the waist circumference and waist-to-hip ratio, the body fat percentage and body adiposity index, as well as the skinfold thickness measurements [29,30].

**Figure 3:**
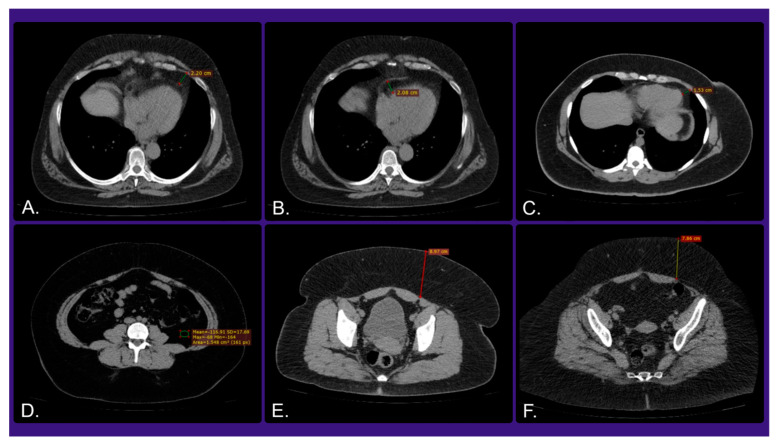
Illustrating computed tomography imaging for the quantification of cardiac and abdominal fat distribution by measuring the thickness of the perivisceral and subcutaneous adipose tissue. Axial sections: (**A**,**C**)—pericardial fat thickness, (**B**)—epicardial fat thickness, (**D**)—abdominal visceral fat density, (**E**,**F**)—thickness of subcutaneous abdominal fat (approval was obtained from the Ethics Committee of the University of Medicine and Pharmacy “Grigore T. Popa” Iasi).

Additionally, the standard paraclinical evaluation of obese patients involves many aspects, such as metabolic profile, inflammatory markers, hormonal and endocrine testing, abdominal imaging (to determine the abdominal fat distribution (Figure 3)), a cardiovascular assessment by ultrasound, and more can follow. Advancements in the cardiac imaging techniques enable the early identification of structural and functional modifications in the hearts of obese patients. The aim of this paraclinical overview of the patient is the comprehension of organ damage dynamics. This subsequently enables an evaluation of how the surgical treatment for obesity affects these variables [31,32,33].

## 4. Cardiovascular Profile After Bariatric Surgery

The cardiovascular implications of BaS among obese patients ought to be closely analyzed, considering their significant long-term impact. Recent studies aim to investigate the postoperative cardiovascular dynamics in obese patients, with the perspective that the management of future BaS candidates could be improved and standardized.

In this regard, a population-based, retrospective cohort study [34] showed significant improvements in BMI, blood pressure, diabetes mellitus, dyslipidemia, metabolic syndrome, lipid profiles, glycated hemoglobin (HbA1c), and liver enzyme levels after BaS. Additionally, reductions in resting heart rate and a shortening of the corrected QT interval were observed on electrocardiograms. The BMI significantly decreased from 45 kg/m^2^ to 32.8 kg/m^2^ within six months, aligning with the results from other studies. Initially, 24% of patients had hypertension, a percentage which decreased by half six months post-surgery. Dyslipidemia was present in 32% of the patients enrolled in the study, also showing significant reductions post-surgery. Moreover, diabetes mellitus was present in 24% of the patients initially, with a 50% remission rate post-surgery. The prevalence of metabolic syndrome decreased by 52% post-surgery, while improvements in lipid profiles were positively concordant with previous research. Also, in this study, the Framingham Risk Score showed a considerable reduction in the 10-year risk span of coronary events, with vascular age also significantly decreasing. That is to say, according to the Ammar et al. study [34], BaS significantly improves cardiovascular risk factors and cardiac function in patients presenting with morbid obesity and related cardiovascular comorbidities, therefore confirming the efficacy of this treatment.

Another study concluded that, while both BaS and a very low-energy-based diet resulted in similar improvements in HbA1c, insulin sensitivity, and blood pressure, BaS led to notably greater results in the lowering of LDL cholesterol levels. This reduction, with a value approximately 18 mg/dL greater in the BaS group, is considered clinically significant and could potentially lead to a reduced risk of cardiovascular disease. The study suggests that approximately half of this LDL cholesterol reduction is a direct effect of the surgical procedure itself rather than due to the loss of body weight [35].

On the other hand, Yuan and colleagues [36] aimed to assess the long-term impact of early RYGB on the incidence of atrial fibrillation (AF) and other major adverse cardiovascular events (MACE). Their cohort study included 1009 patients, with 544 undergoing RYGB during the study period, 308 within one year of the initial consultation (RYGB-1Y), and 701 not within one year (No-RYGB-1Y), the latter group receiving only medical therapy and then “late” RYGB surgery. As a result, the most common comorbidities included hypertension, dyslipidemia, diabetes, and coronary disease, linked to a lower eligibility for early RYGB. The RYGB-1Y group presented higher BMI but lower systolic blood pressure, glucose, HbA1c, and creatinine levels. Initially, there was no substantial difference in the incidence of AF between the groups. However, over a 15-year timespan, the AF incidence was notably lower in the RYGB-1Y group compared to the No-RYGB-1Y group. Furthermore, the RYGB-1Y group revealed remarkably lower risks of mortality, myocardial infarction, heart failure admission, and MACE in contrast with the No-RYGB-1Y group. Patients who underwent early surgery showed a better quality of life compared to those treated solely with medical therapy. These findings suggest a potential long-term protective effect of RYGB against cardiovascular complications and improved survival in obese individuals.

Moreover, a study from 2019 [37] evaluated the frequency of MACE and examined whether the type of BaS—SG versus RYGB—determined the outcome. Thus, the primary focus was on MACE, which included myocardial infarction, acute ischemic heart disease without myocardial infarction, and acute heart failure. Dysrhythmias, excluding premature beats, were also assessed. On these terms, MACE was a rare occurrence, affecting 0.1% of the patients, while dysrhythmias were reported in 3.4% of the cohort. There were 43 recorded deaths, with 31 occurring in patients with MACE or dysrhythmias. Independent risk factors for MACE included an age of 50 years or older, male sex, congestive heart failure, chronic pulmonary disease, ischemic heart disease, a history of pulmonary emboli, and fluid or electrolyte disorders. The type of BaS was, however, not a significant predictor of MACE. Additionally, patients with MACE incurred higher hospital charges, reflecting the increased complexity and resource utilization associated with managing these complications. The study supports the use of RYGB in high-risk patients due to its safety profile and potential benefits in treating severe obesity-related conditions. These findings emphasize the importance of personalized risk assessment in surgical planning. Thorough preoperative evaluations used to identify high-risk patients are essential, suggesting that tailored risk assessment tools for the bariatric population could enhance surgical outcomes and patient safety.

In the same way, another research study concluded that BaS was associated with a significantly lower incidence of MACE in patients with obesity and pre-existing CVD. This suggests a potential protective effect of BaS against further cardiovascular complications in this high-risk population. However, the authors acknowledge the limitations of the retrospective cohort design included in their study and call for a large-scale randomized controlled trial in order to confirm these findings and establish causality [38].

In recent years, numerous studies have specifically analyzed the cardiovascular impact of BaS, with a focus on cardiovascular dynamics, in the management of obese patients undergoing a surgical procedure. We present some of this research in the table below (Table 1).

Overall, the literature describes a great number of cardiovascular markers measured in obese patients before and after BaS. Many of these markers, such as inflammation or atherosclerosis-specific markers, underwent favorable changes following BaS procedures, indicating an improvement in both metabolic status and cardiovascular function in these patients [44].

## 5. Cardiovascular Imaging Outcomes After Bariatric Surgery

Cardiovascular imaging plays an essential role in understanding the dynamics of cardiovascular structure and function in obese patients who have undergone a bariatric procedure.

For example, echocardiographic findings indicate positive correlations between preoperative parameters (e.g., left atrium, aorta, left ventricular (LV) end-systolic diameter, interventricular septum diameter, posterior wall diameter, LV mass index, A wave) and postoperative BMI and negative correlations between the E wave, E/A ratio, and postoperative BMI. Decreases in LV dimensions and mass index along with improved LV ejection fraction and E/A ratio indicate an enhanced cardiac function post-surgery. The positive correlations between the postoperative BMI and the LV mass index as well as the ejection fraction highlight the cardiac benefits of BaS [34].

In addition to this, the carotid intima-media thickness (C-IMT) is a criterion for the diagnosis of recurrent cardiovascular diseases in obese patients. Lunger and colleagues [45] analyzed the C-IMT evolution over a time period of 10 years for patients treated with BaS compared with a matched control group. The maximum C-IMT at the left and right common carotid artery was measured nearby the carotid bulb (the last 2 cm) and then centered to acquire the mean maximum C-IMT. This study revealed the influence of BaS on C-IMT and the metabolism of the cardiovascular system based on 21 patients who underwent bariatric treatment and an additional control group. The results demonstrated that C-IMT remained relatively consistent in the BaS group, while it elevated in the control group, demonstrating the fact that bariatric treatment may avert C-IMT progression. Nevertheless, controls exposed an annual increase of 0.0196 mm as opposed to the surgically treated patient with a rise of 0.0003 mm. This evidence mirrors the benefits of BaS in decreasing the progression of atherosclerosis in the long-run. Notwithstanding, previous studies conclude that there is a link between reduced C-IMT and weight loss, even if it is interventional or non-surgical. Moreover, improving cardiovascular risk factors, such as hypertension, diabetes, and dyslipidemia, positively mediates this effect. The study’s strengths comprised a long observation period and a suitable controlled group. Thus, the results demonstrate the efficacy of BaS in preventing C-IMT progression, highlighting its role in reducing long-term cardiovascular risks.

Moreover, Gluszewska and colleagues [46] analyzed the short- and long-term impacts of BaS on arterial structure and function. They assessed functional vascular changes by measuring the flow-mediated dilation and the nitroglycerin-mediated dilation of the brachial artery. They also measured vascular structural changes as estimated by C-IMT in the short- and medium-term following the bariatric procedure. This study included 71 patients with extreme obesity, and the measurements were performed by ultrasound imaging. In this case, the results showed that the endothelial function and subclinical atherosclerosis improved after BaS. The lack of dilation changes independent of endothelial function seems to suggest the persistence of residual changes at the vascular level. Additionally, C-IMT showed a reduction across the entire population after 6 months, with its baseline values exhibiting a correlation with BMI.

Another noteworthy aspect is the manner in which weight loss affects the cardiac remodeling processes. In this regard, Sorimachi and colleagues [47] investigated the extended effects that substantial weight loss following BaS have on the heart. The research specifically examined changes in epicardial fat (fat surrounding the heart) and abdominal visceral fat, and their respective effects on heart remodeling, particularly in the left ventricle (LV), right ventricle (RV), and left atrium (LA). While the study was ongoing, the 213 patients who underwent BaS showed significant long-term weight loss and improvements in cardiac function. Over a median follow-up of 5.3 years, patients experienced a 23% reduction in body weight and a 22% decrease in BMI. A subset of patients underwent CT scans, which showed a 30% reduction in visceral fat and a 29% decrease in subcutaneous fat along with a 14% reduction in epicardial fat in the broader study group. Echocardiographic assessments revealed significant improvements in both LV and RV functions, with notable reductions in LV mass and wall thickness, linked more to the decrease in visceral fat rather than BMI. Enhanced myocardial mechanics were indicated by improved LV and RV global longitudinal strains. These findings underline the importance of reducing visceral fat for the improvement of cardiac function, suggesting that targeting visceral fat may be more effective in addressing obesity-related cardiac dysfunction than overall weight loss alone. BaS has the potential to induce favorable long-term heart remodeling, with sustained improvements in both LV and RV mechanics.

An additional study by Bucerius et al. [48] examined how BaS has direct implications not only in the vascular atherosclerosis development process, and particularly in the inflammation at the level of the carotid artery, but also in the metabolic activity of other fatty tissues. The participants in this study underwent 18F-fluorodeoxyglucose (18F-FDG) PET/CT scans before and one year after BaS with the purpose of measuring metabolic activity in the carotid arteries, pericardial adipose tissue (PAT), visceral adipose tissue (VAT), and brown adipose tissue (BAT). Before undergoing BaS, obese patients had significantly higher 18F-FDG uptake in their carotid arteries compared to lean and overweight control groups. After surgery, the 18F-FDG uptake markedly decreased, reaching levels similar to those in the control groups. This suggests that carotid artery inflammation normalizes within a year post-surgery. Nevertheless, the metabolic activity in PAT and VAT significantly decreased following BaS. There was a strong positive correlation between the decreases in PAT and VAT activity and the reduction in carotid inflammation. Furthermore, an increase in BAT activity was observed in five out of ten patients post-surgery, which correlated with decreased PAT inflammation. One year after undergoing BaS, there were notable reductions in inflammation of the carotid artery and metabolic activity in both PAT and VAT. The activity of BAT also increased, indicating an improved metabolic state. The relationship observed between reduced carotid inflammation and decreased VAT activity highlights the connection between the inflammation of fatty tissue and vascular health. The benefits of BaS on vascular health, therefore, seem to extend beyond mere weight loss, suggesting it also has direct anti-inflammatory effects. A significant relationship was found between changes in subcutaneous adipose tissue (SAT) activity and improvements in carotid inflammation, PAT, and VAT. This supports the notion that VAT poses a higher cardiovascular risk than SAT. This study reinforces the extensive benefits of BaS, extending beyond weight loss, including reduced inflammation and improved metabolic health.

## 6. Discussion

Overall, obesity remains a particularly severe chronic condition with a large variety of systemic implications and increased morbidity. The prevalence of obesity has risen dramatically in recent decades, with alarmingly high rates among both adults and children. Addressing the obesity epidemic has become a pressing public health concern, with bariatric procedures emerging as a crucial therapeutic intervention for individuals struggling with severe obesity.

Indeed, obesity has various severe clinical implications, and, more specifically, in terms of its effects on the cardiovascular profile, it is linked to changes in cardiac structure and hemodynamics, which further contribute to increased cardiovascular morbidity and mortality. In this regard, BaS has refined the therapeutic approach to morbid obesity. Emerging evidence indicates that BaS leads to improvements in both cardiac structure and function [49].

As for the cardiovascular complications following BaS, though rare, they prove to exert a great influence on the short- and long-term evolution of the patients, with significant increases in hospital stay, costs, and mortality. Age, sex, and several comorbidities, such as type 2 diabetes, obstructive sleep apnea, or non-alcoholic fatty liver disease, are significant predictors of these events. However, the specific type of BaS procedure does not independently affect the risk of MACE. These insights can be a useful tool in guiding better patient selection as well as in asserting preoperative potential risks, therefore improving surgical outcomes and patient counseling [50,51].

In this regard, our research also inquired into particular cardiac complications following BaS. For example, ongoing LA dysfunction despite significant weight loss suggests the necessity for continuous monitoring and potentially targeted interventions to address this specific issue. The resulting clinical implications advocate for comprehensive weight loss strategies that expressly target visceral fat in order to enhance cardiac outcomes in obese patients. However, the persistence of LA dysfunction highlights the need for further research and better targeted interventions. The findings emphasize the necessity of prospective randomized controlled trials in order to confirm these observations and explore additional weight loss strategies for the optimal management of obesity-related cardiac dysfunction [47].

In light of the evidence from other recent studies, we have a better understanding of the significant improvements in LVEF as well as a clearer perspective of the LV morphologic parameters after BaS. Thus, BaS was notably associated with a 25–50% reduction in the risk of the composite outcome of postoperative myocardial infarction, stroke, or death compared to other morbidly obese surgical patients [8,52].

Another intriguing aspect resides in the fact that a negative correlation was noted between increased BAT activity and decreased carotid inflammation, indicating potential anti-inflammatory effects of BAT on blood vessels, which is further supported by the association between increased BAT activity and reduced PAT activity. For this reason, the increase in BAT activity post-surgery represents a potential target for the further improvement of metabolic health and for the reduction in the cardiovascular risk. Future research ought to investigate the mechanisms of BaS and the role of BAT, with larger studies being required in order to confirm these findings [48].

One of the most recent studies [53] provides compelling evidence that weight loss can result in the reverse of cardiac remodeling and, therefore, improves cardiac structure and function. BaS is widely regarded as the most effective and sustainable long-term intervention for achieving significant weight loss in patients with severe obesity. Beyond mitigating metabolic syndrome, numerous studies have demonstrated the positive effects of BaS on cardiac structure and function, confirming the positive results.

There is widespread consensus and substantial research indicating that obesity carries considerable health consequences. Excessive body weight beyond the normal range is closely correlated with the emergence of various risk factors and an elevated incidence of cardiovascular morbidity and mortality. Multiple factors can be measured to assess the cardiovascular effects of BaS. A comprehensive cardiovascular assessment is essential to quantify the pre- and post-surgical status of the obese patient.

To a large extent, our findings indicate that BaS is linked with an overall reduced cardiovascular mortality as well as a low incidence of cardiovascular disease when used as the preferred therapy for patients with obesity.

## 7. Conclusions

In conclusion, the global increase in obesity represents a significant public health challenge, leading to higher mortality and, subsequently, to majorly elevated healthcare costs. BaS is a highly effective treatment, performed on obese patients in order to obtain significant and sustained weight loss as well as with the aim of improving associated co-diagnoses. Without a doubt, the results of BaS involve a significant amelioration of the BMI, contributing to a considerable decrease in cardiovascular risk factors and to a notable refinement in the cardiovascular structure and function, making it an effective intervention for morbid obesity and related comorbidities.

## Figures and Tables

**Table 1:** Studies investigating the cardiovascular outcomes of BaS.

Study	Purpose	Number of Patients	Results
Hughes et al., 2024 [39]	Examining the changes in cardiac structure and function following BaS, with a specific focus on LV global longitudinal strain	398	BaS demonstrated beneficial effects on LV structure and function. However, despite these improvements, obesity and LV hypertrophy persisted in many patients at the time of follow-up.
Xu et al., 2023 [40]	Evaluating the effect of BaS on the CVD risk within the Chinese population	61	Patients with obesity exhibited significantly reduced CVD risk following BaS.
Näslund et al., 2021 [41]	The analysis of the relationship between BaS and the occurrence of MACE in individuals with a history of MI and severe obesity	1319	BaS has been found to be strongly associated with a reduced incidence of severe complications in individuals with a history of MI and severe obesity. Specifically, this procedure is linked to a lower occurrence of MACE and a decrease in overall mortality. Additionally, BaS is correlated with a reduced risk of recurrent MI and the development of new-onset HF in this patient population.
Moussa et al., 2020 [42]	Assessing the long-term impact of BaS on cardiovascular outcomes in patients with obesity	3701	BaS has been shown to reduce the long-term risk of severe cardiovascular events in individuals with obesity. Moreover, it is associated with a decreased incidence of acute HF in this population over time.
Benotti et al., 2017 [43]	The association between BaS and long-term cardiovascular events	1724	RYGB is linked to a decreased risk of MACE and a lower likelihood of developing congestive HF.

BaS: bariatric surgery; CVD: cardiovascular disease; HF: heart failure; LV: left ventricle; MACE: major adverse cardiovascular events; MI: myocardial infarction; RYGB: Roux-en-Y gastric bypass.

## Data Availability

Not applicable.
